# A single-center experience with microbiologic surveillance of LivaNova 3T heater-cooler devices (HCDs)

**DOI:** 10.1017/ash.2023.356

**Published:** 2023-09-29

**Authors:** Scott Curry, Yosra Alkabab, Danny Nixon, Susan Dorman, Cassandra Salgado

## Abstract

**Background:** The global outbreak of *Mycobacterium chimaera* infections associated with HCDs resulted in new maintenance recommendations. Since 2018, HCDs have been disinfected according to instructions for use (IFU), including twice-monthly bleach disinfection and monitoring hydrogen peroxide (H_2_O_2_) to maintain a minimum daily concentration of 100 ppm. In February 2020, the IFU added the recommendation to perform microbiologic surveillance of HCD tank water to ensure effectiveness of disinfection to levels of <1 colony forming unit per milliliter (CFU/mL) of nontuberculous mycobacterium (NTM). We report our experience with this microbiologic surveillance as well as that of culturing the HCD environment to investigate modes of transmission. **Methods:** In 2022, we began culturing tank water in 10 HCDs for NTM. For a subset of 6 HCDs, quantitative NTM culturing of tank water before and after bleach disinfection was done. After initial results indicated widespread-contamination of HCDs with *M. chimaera*, we performed fill water cultures from 5 sinks in 4 HCD maintenance rooms. We also conducted 20 two-hour NTM settle-plate cultures of a cardiac operating room (OR) at different sites both inside (n = 7) and outside (n = 3) the OR: 10 with the HCD (located outside the OR) turned off (controls) and 10 with HCD turned on (exposure). A paired *t* test was used to evaluate differences in mean recovery of NTM in tank water samples. **Results:** Cultures from 7 (70%) of 10 HCDs were positive, with a mean of 13.6 CFU/mL *M. chimaera* (Table 1). There was no significant difference between the 10 pairs of pre- and postdisinfection NTM cultures done according to the IFU from 6 HCDs: mean predisinfection cultures (15.5 CFU/mL) versus mean postdisinfection cultures (12 CFU/mL) (*P* = .90) (Table 2). For fill water, 1 of 7 sink samples in 1 of 4 rooms was positive for *M. chimaera* ( <1 CFU/mL) from a specimen from a fresh 0.2-µm filter that had been stored in the fill-sink splash zone. OR settle-plate cultures showed 0 (0%) of 10 control sites and 1 (10 %) of 10 exposure sites inside the OR positive for NTM, with a single CFU of *M. avium–intracellulare* complex. **Conclusions:** Our data cannot clearly refute either of 2 possible scenarios for HCD contamination: cross contamination during device maintenance versus at the point of manufacture. Despite the IFU guidance or disinfection being implemented, disinfection procedures failed to suppress NTM contamination, and tank water within most HCDs was contaminated with *M. chimaera* regardless of age or whether it was deep cleaned or upgraded with an aerosol containment device.

**Figure:**
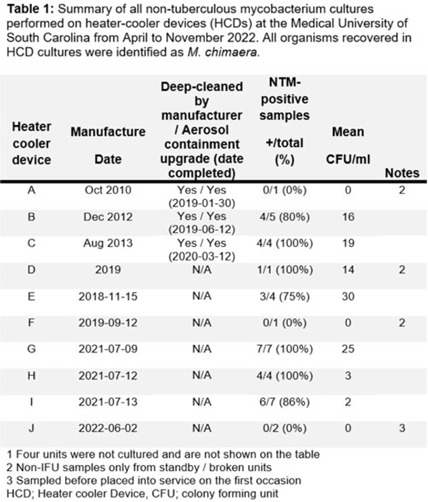

**Figure:**
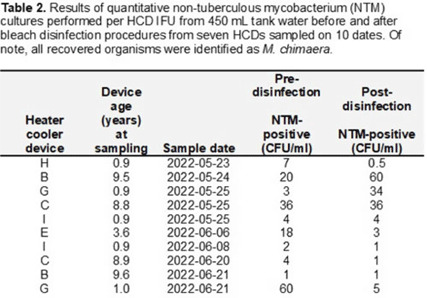

**Disclosures:** None

